# Magnetic fields as biophysical activators of autophagy: A preclinical systematic review

**DOI:** 10.1016/j.bbrep.2026.102613

**Published:** 2026-05-01

**Authors:** Enzo Emanuele, Alejandro Santos-Lozano, Susana López-Ortiz, Kayvan Khoramipour, Celia García-Chico, Simone Lista, Piercarlo Minoretti

**Affiliations:** a 2E Science, Robbio, Pavia, 27038, Italy; b i+HeALTH Strategic Research Group, Department of Health Sciences, Miguel de Cervantes European University (UEMC), Valladolid, 47012, Spain; c Studio Minoretti, Oggiono, Lecco, 23848, Italy; d Department of Social Sciences, Miguel de Cervantes European University (UEMC), Valladolid, 47012, Spain

**Keywords:** Magnetic fields, Autophagy, Preclinical models, Repetitive transcranial magnetic stimulation, Neurodegenerative diseases, Cancer

## Abstract

While pharmacological inducers of autophagy have been extensively studied, their systemic adverse effects may limit clinical use. Increasing preclinical evidence suggests that magnetic fields (MFs) can represent a non-invasive alternative for autophagy modulation. In this systematic review, we sought to evaluate preclinical evidence on MF-mediated autophagy activation, including intervention parameters, mechanistic pathways, and therapeutic outcomes, to guide future translational research. Following PRISMA guidelines, we searched (January 2010–August 2025) four structured electronic databases (PubMed, Scopus, Embase, and IEEE Xplore) for studies investigating MF effects on autophagy in preclinical models. Eligible reports included *in vitro* and animal investigations with clearly defined MF parameters and validated autophagy markers. Nine studies met inclusion criteria, covering diverse MF modalities such as repetitive transcranial magnetic stimulation, rotating fields, pulsed and power-frequency fields, and radiofrequency exposures. Across a wide range of frequencies (1–50 Hz) and intensities (20 mT–1.5 T), most studies reported autophagy activation, as evidenced by LC3-II accumulation, Beclin-1 upregulation, p62 degradation, and autophagic flux confirmation in select experimental models. Mechanistic analyses converged on PI3K/AKT/mTOR inhibition. Functionally, MF-induced autophagy conferred neuronal protection and drove behavioral recovery in models of Alzheimer's disease, vascular dementia, and stress, whereas it triggered autophagic cell death in cancer. Some studies reported incomplete flux or autophagosome accumulation. Risk of bias was generally unclear due to methodological heterogeneity. In summary, preclinical evidence indicates that MFs can act as versatile, non-pharmacological activators of autophagy. Defining optimal stimulation parameters, clarifying mechanistic pathways, and advancing translational studies will be essential for clinical application.

## Highlights

• Magnetic fields activate autophagy in diverse preclinical systems.
• Convergent inhibition of the PI3K-AKT-mTOR pathway supports flux.
• CNS disease models show cognitive gains with autophagy induction.
• Oncology models reveal autophagy-associated tumor cell death.
• Effects depend on parameters and some contexts show incomplete flux.

## 1 Introduction

Disorders characterized by impaired cellular quality control mechanisms – including neurodegenerative diseases [1] and various malignancies [2,3] – collectively affect hundreds of millions of patients worldwide and represent some of medicine's greatest therapeutic challenges [4,5]. Central to the pathogenesis of these conditions is defective autophagy – an evolutionarily conserved cellular degradation pathway that removes damaged proteins and organelles while providing metabolic substrates during cellular stress [6,7]. The accumulation of toxic protein aggregates in neurodegeneration [8], the metabolic reprogramming that enables cancer cell survival [9], and the cellular dysfunction underlying aging-related diseases [10] all reflect, at least in part, inadequate autophagic clearance. In addition, impaired autophagy is universally recognized as a major hallmark of aging [11]. Beyond lifestyle and dietary interventions such as physical exercise [12] and caloric restriction [13], current strategies to induce autophagy rely primarily on pharmacological agents, including rapamycin (and rapalogs) [14,15], metformin [16], and various natural compounds such as trehalose [17] and resveratrol [18]. However, pharmacological inducers of autophagy are frequently associated with systemic adverse effects that significantly limit their clinical applicability, particularly in vulnerable populations such as elderly individuals with neurodegenerative disorders [19]. Moreover, the complex, context-dependent nature of autophagy regulation makes it challenging to achieve therapeutically meaningful activation without disrupting normal cellular homeostasis [20].

In recent years, application of magnetic fields (MFs) has gained recognition as a promising non-invasive strategy for modulating numerous cellular processes [[21], [22], [23], [24], [25]]. For instance, repetitive transcranial magnetic stimulation (rTMS) – already established in clinical practice for unipolar major depression [26] and other psychiatric and neurological diseases [27] – illustrates how precisely controlled MFs can modulate cellular signaling pathways while producing minimal systemic toxicity. Notably, the ability to fine-tune MF parameters – including frequency, intensity, and duration of stimulation [28] – may theoretically offer unprecedented precision for therapeutic interventions, potentially overcoming the limitations of systemic drug therapy. In this regard, recent research has begun to investigate whether MFs can regulate fundamental cellular maintenance pathways, including autophagy. This approach is supported by evidence that application of MFs can modify intracellular signaling cascades, including the mechanistic target of rapamycin (mTOR) pathway [29], which serves as the primary negative regulator of autophagy [30]. If MFs can consistently stimulate autophagy across various disease contexts, they can theoretically offer a paradigm shift in the management of disorders linked to defective cellular quality control, delivering targeted benefits without the systemic drawbacks of pharmacological interventions. We therefore conducted a systematic review of the published literature on MF-mediated autophagy activation in preclinical models, aiming to summarize intervention parameters, elucidate underlying mechanisms, evaluate therapeutic outcomes, and provide guidance on clinical translation and future research directions.

## 2 Materials and methods

This preclinical systematic review was conducted in accordance with the Preferred Reporting Items for Systematic Reviews and Meta-Analyses (PRISMA) guidelines [31].

### 2.1 Eligibility criteria

The Population, Intervention, Comparison, Outcomes, and Study design (PICOS) framework was used to define eligibility criteria [32]. Eligible studies included preclinical models (*in vitro*, *in vivo*, and *ex vivo*) evaluating the effects of MFs on autophagy and associated pathways. Interventions comprised MF applications of any modality [33], including rTMS, pulsed electromagnetic fields, static MFs, rotating MFs, and radiofrequency electromagnetic fields, provided that field parameters – including frequency, intensity, and exposure duration – were clearly specified. Comparison required studies to incorporate appropriate control conditions with sham or vehicle treatments applied using identical protocols to the MF interventions. Outcome measures included primary assessment of established autophagy markers – including LC3-I/II conversion, p62/SQSTM1 degradation, Beclin-1 expression, or other ATG proteins, along with functional measures of autophagic activity, while secondary outcomes comprised mechanistic pathway analysis [34]. Eligible studies were quantitative, peer-reviewed investigations published in English-language journals. Studies were excluded if they were non-original research (reviews, abstracts, conference proceedings), lacked appropriate control groups, failed to assess autophagy markers or autophagic flux, or provided insufficient detail regarding MF parameters.

### 2.2 Search strategy

A literature search was conducted across four structured electronic databases (PubMed, Scopus, Embase, and IEEE Xplore), covering original research articles from January 2010 through August 2025. Google Scholar was additionally used for forward and backward citation tracking to identify potentially relevant records not captured by the primary database searches. The complete search string was as follows: (“magnetic field” OR “magnetic fields” OR “electromagnetic field” OR “electromagnetic fields” OR “transcranial magnetic stimulation” OR “pulsed electromagnetic field” OR “pulsed electromagnetic fields” OR “rotating magnetic field” OR “rotating magnetic fields” OR “static magnetic field” OR “static magnetic fields” OR “radiofrequency electromagnetic field”) AND (“autophagy” OR “autophagic” OR “autophagosome” OR “autophagosomes” OR “LC3” OR “Beclin” OR “Beclin-1” OR “ATG” OR “ATG5” OR “ATG7” OR “mTOR pathway” OR “autophagic flux” OR “p62” OR “SQSTM1”). Equivalent search strategies, adapted to the specific syntax requirements of each database, were employed for Scopus, Embase, and IEEE Xplore. Reference lists of relevant studies were manually examined to identify additional eligible publications. Two authors conducted all searches independently.

### 2.3 Study selection

Two investigators (E.E. and P.M.) independently screened all retrieved articles. The screening process involved initial title and abstract evaluation according to the predetermined eligibility criteria, followed by full-text assessment of potentially relevant studies. Any disagreements between reviewers were resolved through discussion, with consultation of a third reviewer (S.L.) when consensus could not be reached. Deduplication of retrieved records and management of the screening workflow were performed using Microsoft Excel spreadsheets.

### 2.4 Data extraction

A comprehensive data extraction protocol was developed to systematically capture key information including study characteristics (authorship, publication year, experimental design), MF specifications (exposure protocols, modality, frequency, intensity, duration), experimental model details (*in vitro*, *in vivo*, and *ex vivo*), autophagy assessment methods (Western blotting techniques, microscopy approaches, flux assays), outcome measures, mechanistic insights, and therapeutic implications. Two reviewers (E.E. and P.M.) independently performed data extraction using standardized forms, with discrepancies addressed through collaborative review and consensus.

### 2.5 Quality assessment

Animal study quality was assessed using the SYRCLE risk-of-bias tool [35], adapted from the Cochrane framework, which evaluates the following ten domains: sequence generation, baseline characteristics, allocation concealment, random housing, blinding of caregivers and investigators, random outcome assessment, blinding of outcome assessors, incomplete outcome data, selective outcome reporting, and other sources of bias. Each domain was rated as low, high, or unclear risk of bias.

### 2.6 Data synthesis and analysis

Given the substantial heterogeneity in MF parameters, experimental methodologies, and outcome measurements, a qualitative synthesis approach was employed. Studies were systematically categorized by MF modality and evaluated for consistent patterns of autophagy modulation. Mechanistic pathways were analyzed across studies to identify convergent molecular targets and regulatory mechanisms. A formal meta-analysis was not possible due to methodological diversity across the included studies.

## 3 Results

### 3.1 Study selection

**Fig. 1:**
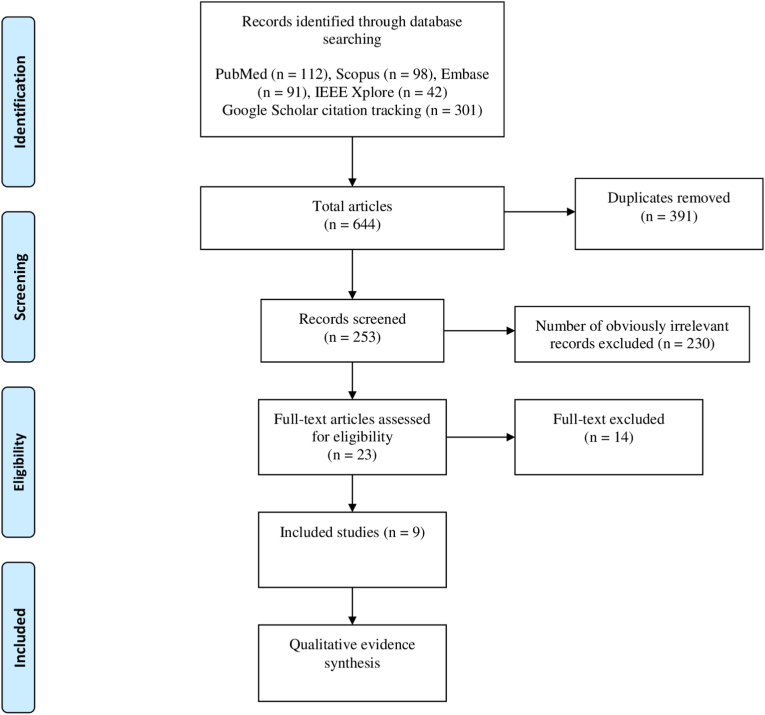
PRISMA flow diagram.

The systematic search identified nine preclinical studies that met the inclusion criteria – comprising both *in vitro* cellular systems and *in vivo* animal disease models. The PRISMA flow diagram detailing the selection process is shown in Fig. 1.

### 3.2 Study characteristics

Table 1 summarizes the preclinical studies investigating the effects of MFs on autophagy. Cellular models included mouse embryonic fibroblasts [42], human bone mesenchymal stromal cells [41], SH-SY5Y neuroblastoma cells [43], N9 microglia [43], primary astrocytes [39], and HT22 hippocampal neurons [29]. Animal models comprised APP/PS1 Alzheimer's disease (AD) mice [29], two-vessel occlusion rats [37], spinal cord injury mice [39], chronic unpredictable mild stress (CUMS) mice [38], and Lewis lung carcinoma models [40]. MF modalities were as follows: five studies evaluated rTMS (frequency: 1–50 Hz, intensity: 20 mT–1.5 T) [[36], [37], [38], [39],^41^], one investigated a power-frequency field (frequency: 50 Hz, intensity: 2 mT) [42]; one tested a low-frequency anti-tumor field (frequency: 7.5 Hz, intensity: 0.4 T) [40]; one applied a long-term rotating MF (frequency: 4 Hz, intensity: 0.2 T) [29]; and one employed extremely low frequency (ELF)-modulated radiofrequency (RF) fields (frequency: 935 MHz, specific absorption rate [SAR]: 4 W/kg) [43]. Autophagy endpoints were assessed using LC3-II [39] or LC3-II/I ratios [36,41], p62 [36,37,39,41], Beclin-1 and Atg7 expression [37,38], ATG5 levels [43], GFP-LC3 puncta formation [42], autophagic vacuoles by electron microscopy [42], flux analysis [41,42], and interrogation of signaling pathways such as PI3K/AKT/mTOR [29,36,[39], [40], [41]], ERK [41,43], Ca^2+^ [41], and reactive oxygen species (ROS) [42].

**Table 1:** Summary of the included studies.

Study	MF modality	Frequency	Intensity	Exposure protocol	Model system	Autophagy markers	Use of gold-standard flux assays	Key pathway	Therapeutic outcome
[36]	rTMS (HF)	5 Hz	Intensity reported as 120% of resting motor threshold; absolute field strength in Tesla not stated in the original publication	14 days	APP/PS1 mice (*in vivo*)	LC3-II/I, p62	No (autophagy markers determined by Western blot)	ApoE reduction	Cognitive improvement
[29]	Rotating MF	4 Hz	0.2 T	2 h/day, 6 months	APP/PS1 mice; HT22 cells	Autophagy panel; PI3K/AKT/mTOR	No (autophagy markers determined by immunofluorescence and Western blot)	PI3K/AKT/mTOR inhibition	Cognitive improvement
[37]	rTMS	5 Hz	0.5 T	+/− MSC transplantation	Vascular dementia rats (*in vivo*)	LC3-II, Beclin-1, p62	No (autophagy markers determined by immunofluorescence and Western blot)	Autophagy upregulation; synergistic with MSCs	Synaptic plasticity; cognitive gains
[38]	LF-TMS	1 Hz	20 mT	1 min/day, 28 days	CUMS mice (*in vivo*)	Atg7, Beclin-1, *p*-mTOR/mTOR	No (autophagy markers determined by immunostaining and Western blot)	mTOR normalization	Behavioral recovery
[39]	rTMS (HF)	15 Hz	0.5 T	6 weeks *in vivo*	SCI mice; astrocytes	LC3-II, p62, mTOR	No (autophagy markers determined by immunofluorescence and Western blot)	Cx43-autophagy loop; mTOR activation	Motor function recovery
[40]	Low-frequency anti-tumor MF	7.5 Hz	0.4 T	4 h/day, 35 days	Lewis lung carcinoma (*in vivo*)	LC3-II, Atg5 and Beclin-1	No (autophagy markers determined by immunostaining and Western blot)	miR-486/BCAP/AKT/mTOR	Tumor growth inhibition
[41]	rTMS	50 Hz	0.5 T (1.0/1.5 T ineffective)	20 min/day, up to 5 days	Human bone MSCs (*in vitro*)	LC3-II/I, p62, Ca2+, *p*-ERK, *p*-mTOR	Yes (3BDO-controlled experiments)	NMDAR-Ca^2+^-ERK/mTOR	N/A (*in vitro*)
[42]	Power-frequency MF	50 Hz	2 mT	0.5-24 h	Mouse embryonic fibroblasts (*in vitro*)	LC3-II, GFP-LC3, TEM, flux with CQ	Yes (CQ-controlled experiments)	ROS; mTOR-independent	N/A (*in vitro*)
[43]	ELF-modulated RF-EMF	935 MHz (SAR 4 W/kg)	N/A (SAR-based)	2 h and 24 h	SH-SY5Y; N9 microglia (*in vitro*)	ATG5, LC3B–I/II, *p*-ERK	No (autophagy markers determined by Western blot)	No significant pathway engagement	N/A (*in vitro*)

Abbreviations: 3BDO, 3-benzyl-5-[(2-nitrophenoxy)methyl]-2-oxazolidinone; AKT, protein kinase B; ApoE, apolipoprotein E; APP/PS1, amyloid precursor protein/presenilin 1; ATG5, autophagy related 5; ATG7, autophagy related 7; BCAP, B-cell receptor-associated protein; Beclin-1, Beclin 1; Ca^2+^, calcium ion; CQ, chloroquine; CUMS, chronic unpredictable mild stress; Cx43, connexin 43; ELF, extremely low frequency; ERK, extracellular signal-regulated kinase; GFP, green fluorescent protein; HF-rTMS, high-frequency repetitive transcranial magnetic stimulation; HT22, mouse hippocampal neuronal cell line; Hz, Hertz; LC3, microtubule-associated protein 1A/1B-light chain 3; LC3-II, LC3-phosphatidylethanolamine conjugate; LC3B, microtubule-associated protein 1A/1B-light chain 3B; LF-TMS, low-frequency transcranial magnetic stimulation; MF, magnetic field; miR-486, microRNA-486; MSCs, mesenchymal stromal cells; mTOR, mechanistic target of rapamycin; N9, mouse microglial cell line; NMDAR, N-methyl-d-aspartate receptor; *p*-ERK, phosphorylated ERK; *p*-mTOR, phosphorylated mTOR; p-S6, phosphorylated ribosomal protein S6; p62, sequestosome 1; PI3K, phosphoinositide 3-kinase; RF-EMF, radiofrequency electromagnetic field; ROS, reactive oxygen species; rTMS, repetitive transcranial magnetic stimulation; SAR, specific absorption rate; SCI, spinal cord injury; SH-SY5Y, human neuroblastoma cell line; T, Tesla; TEM, transmission electron microscopy.

### 3.3 Magnetic field-induced autophagy activation

Several MF paradigms were found to activate canonical autophagic flux, typically reflected by increased LC3-II or LC3-II/I ratios with concomitant p62 degradation. In human bone mesenchymal stromal cells, Wang et al. [41] reported that 50-Hz rTMS at 0.5 T increased the LC3-II/I ratio and reduced p62 through NMDA receptor-Ca^2+^-ERK signaling with mTOR inhibition. Similar findings emerged using *in vivo* animal models. Accordingly, high-frequency rTMS increased the LC3-II/I ratio and reduced p62 in APP/PS1 AD mice [36]. Similarly, in rats with vascular dementia, 5-Hz rTMS upregulated LC3-II and Beclin-1 and decreased p62, with stronger effects when combined with mesenchymal stromal cell transplantation [37]. Further evidence for flux activation was provided by Chen et al. [42], who showed in mouse embryonic fibroblasts that 50-Hz, 2-mT fields induced LC3-II accumulation, GFP-LC3 puncta, and autophagic vacuoles confirmed by electron microscopy. Flux blockade with chloroquine revealed a ROS-dependent but mTOR-independent pathway of regulation. In stressed mice, low-frequency TMS at 1 Hz successfully restored Atg7 and Beclin-1 expression and reversed CUMS-induced suppression of autophagy [38]. In addition, 6-month rotating magnetic field exposure (frequency: 4 Hz, intensity: 0.2 T) in APP/PS1 mice induced the expression of autophagy markers, inhibited PI3K/AKT/mTOR signaling, and was associated with improved cognition and reduced amyloid burden; similar effects were confirmed in Aβ-treated hippocampal neurons [29].

### 3.4 Divergent or incomplete responses

Not all models demonstrated productive autophagic flux. In spinal cord injury astrocytes, Zhang et al. [39] found that high-frequency rTMS (frequency: 15 Hz, intensity: 0.5 T) increased LC3-II in parallel with p62 and was accompanied by mTOR and S6 phosphorylation, a profile consistent with autophagosome accumulation and impaired degradation rather than complete flux. Similarly, Zielinski et al. [43] reported that exposure to ELF-modulated RF-EMF (frequency: 935 MHz, SAR 4 W/kg) modestly increased ATG5 expression after 24 h in neuroblastoma and microglial cells, but did not alter LC3 processing or ERK activation, suggesting limited engagement of autophagy pathways without effective flux progression.

### 3.5 Cancer models

In a Lewis lung carcinoma model, Xu et al. [40] showed that low-frequency MFs (frequency: 7.5 Hz, intensity: 0.4 T) triggered autophagy-associated cell death through a miR-486-AKT/mTOR signaling cascade. In this context, autophagy served as a cytotoxic rather than cytoprotective mechanism, underscoring the disease-specific consequences of MF exposure.

### 3.6 Mechanistic pathways

The nine reviewed studies pointed to diverse but converging mechanisms by which MFs can regulate autophagy. In human bone mesenchymal stromal cells, verified flux was mediated by NMDA receptor-Ca^2+^-ERK activation with mTOR inhibition [41], whereas in mouse embryonic fibroblasts, LC3 lipidation was driven by ROS in an mTOR-independent manner [42]. The Lewis lung carcinoma model engaged a distinct microRNA-regulated axis, in which miR-486 suppressed BCAP to inhibit AKT/mTOR and promote autophagy-associated death [40]. By contrast, in AD [29] and CUMS [38] paradigms, rotating or low-frequency stimulation consistently reduced PI3K/AKT/mTOR phosphorylation, restoring Atg7, Beclin-1, and LC3 turnover. In spinal cord injury, however, rTMS increased LC3 and p62 together with mTOR activation [39], suggesting impaired flux and injury-specific dysregulation.

### 3.7 Therapeutic implications

Across neurodegenerative and psychiatric models, MFs-induced autophagy activation was significantly associated with functional recovery. In APP/PS1 mice [29,36] and vascular dementia rats [37], rTMS [36] or rotating MF stimulation [29] promoted autophagy activation which was paralleled by cognitive gains. Similarly, in CUMS mice, low-frequency TMS restored Atg7 and Beclin-1 expression and reversed synaptic and behavioral deficits [38]. By contrast, in Lewis lung carcinoma, MF-induced autophagy acted as a tumor cell death mechanism, with activation downstream of miR-486-AKT/mTOR suppression [40].

### 3.8 Parameter dependence

MF effects on autophagy were regimen-, intensity-, and frequency-dependent. In human mesenchymal stromal cells, Wang et al. [41] demonstrated a non-linear threshold, with 0.5 T rTMS inducing flux, whereas higher intensities (1.0–1.5 T) were ineffective. Chen et al. [42] showed a time-dependent response in mouse fibroblasts, with LC3-II accumulation peaking at 6 h under 50-Hz, 2-mT fields. Zielinski et al. [43] reported that 24 h of ELF-modulated RF-EMF exposure increased ATG5 but failed to drive LC3 conversion. *In vivo*, both relatively short rTMS courses (two weeks in APP/PS1 mice) [36] and prolonged rotating magnetic field exposures (six months in APP/PS1 mice) [29] stimulated autophagy alongside behavioral improvement, indicating efficacy with both acute and chronic paradigms. Even low-intensity stimulation (frequency: 1 Hz, intensity: 20 mT) restored autophagy markers in stress-suppressed mice [38], indicating that therapeutic effects do not necessarily require high-amplitude input.

### 3.9 Risk of bias assessment

Overall, the quality of *in vivo* studies was judged to be of uncertain risk of bias under the SYRCLE tool. A domain-by-domain evaluation is presented in Table 2. Across the six studies with *in vivo* components, sequence generation was rated as low risk in only one study [38], which explicitly described random group assignment. The remaining five [29,36,37,39,40] were rated as unclear owing to insufficient description of randomization procedures. Baseline characteristics were consistently at low risk, as all studies used age-matched animals of the same strain or genotype. Allocation concealment was unclear in all six studies. Random housing was similarly unreported. Blinding of caregivers and investigators was rated as low risk in two studies [29,39] that described proper sham-control procedures, and unclear in the remainder. Random outcome assessment was rated as low only for Zhang et al. [39], which reported blinded behavioral scoring. Blinding of outcome assessors for biochemical endpoints was unclear across all studies. Incomplete outcome data were at low risk throughout, with no evident unexplained dropouts. Selective outcome reporting was at low risk in four studies and unclear in two [36,40]. Wang et al. [37] received a high risk rating for other sources of bias due to mesenchymal stromal cell co-transplantation, which confounds attribution of autophagy effects to MF stimulation alone.

**Table 2:** SYRCLE risk of bias assessment for *in vivo* studies.

Study	Sequence generation	Baseline characteristics	Allocation concealment	Random housing	Blinding of caregivers	Random outcome assessment	Blinding of assessors	Incomplete outcome data	Selective reporting	Other sources of bias
[36]	Unclear	Low	Unclear	Unclear	Unclear	Unclear	Unclear	Low	Unclear	Unclear
[38]	Low	Low	Unclear	Unclear	Unclear	Unclear	Unclear	Low	Low	Low
[37]	Unclear	Low	Unclear	Unclear	Unclear	Unclear	Unclear	Low	Low	High
[39]	Unclear	Low	Unclear	Unclear	Low	Low	Unclear	Low	Low	Low
[29]	Unclear	Low	Unclear	Unclear	Low	Unclear	Unclear	Low	Low	Unclear
[40]	Unclear	Low	Unclear	Unclear	Unclear	Unclear	Unclear	Low	Unclear	Unclear

Wang et al. [37] received a “High” rating for “Other sources of bias” due to the use of mesenchymal stromal cell co-transplantation, which confounds attribution of autophagy effects to MF stimulation alone. Liu et al. [38] was the only study to explicitly describe random group assignment (“Low” for sequence generation). Zhang et al. [39] and Li et al. [29] were the only studies rated as “Low” for blinding of caregivers; Zhang et al. [39] was additionally the only study reporting blinded behavioral scoring (“Low” for random outcome assessment). No study addressed allocation concealment or random housing.

## 4 Discussion

In this preclinical systematic review, we found consistent evidence that MFs can stimulate autophagy across diverse cellular and animal models, although the underlying mechanisms and optimal stimulation parameters remain heterogeneous. One of the most striking findings from the reviewed literature was the broad range of MF regimens capable of activating autophagy. Reported effective protocols included frequencies from 1 to 50 Hz [38,41,42], intensities between 20 mT and 1.5 T [[38], [39], [40], [41]], and exposure durations spanning from minutes to several months [29,36,41]. While such variability might initially appear to hinder standardization, it more likely reflects the intrinsic sensitivity of the autophagic machinery to MF modulation. The finding that both short, high-intensity stimulation [41] and prolonged, low-intensity exposure [29,38] can activate autophagy suggests that regulation occurs across multiple biophysical windows rather than through a simple linear dose-response. This flexibility underscores the therapeutic promise of MF-based interventions, while also highlighting the complexity of translating preclinical protocols into consistent clinical applications.

**Fig. 2:**
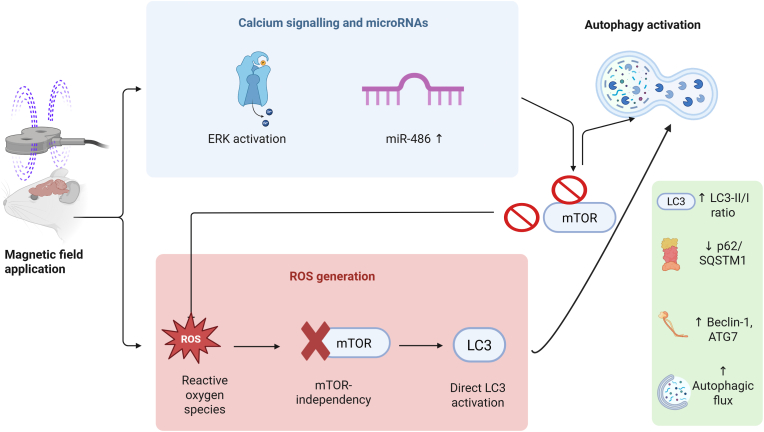
Proposed mechanistic pathways of magnetic field-induced autophagy activation. The upper pathway demonstrates calcium signaling and microRNA regulation. Specifically, magnetic fields activate extracellular signal-regulated kinase (ERK) through calcium-dependent mechanisms, while simultaneously upregulating miR-486. The miR-486 upregulation leads to downstream mechanistic target of rapamycin (mTOR) inhibition through intermediate steps (indicated by prohibition symbols). The lower pathway illustrates reactive oxygen species (ROS)-mediated autophagy induction: magnetic field exposure generates reactive oxygen species, which directly activate LC3 (microtubule-associated protein 1A/1B-light chain 3) in an mTOR-independent manner, bypassing traditional mTOR regulatory mechanisms. Both pathways converge on autophagy activation (right panel), characterized by increased LC3-II/LC3-I ratios, decreased p62/SQSTM1 levels, upregulated Beclin-1 and ATG7 expression, and enhanced autophagic flux. The mechanistic diversity suggests that magnetic fields can modulate autophagy through multiple biophysical windows, supporting their therapeutic potential as non-pharmacological autophagy activators across various disease contexts. Arrows indicate activation or upregulation; prohibition symbols indicate inhibition or downregulation. *Created in BioRender. Santos-Lozano, A. (2025)*https://BioRender.com/opk9tls.

Despite this heterogeneity, several convergent molecular pathways emerged as key mediators of MF-induced autophagy. The PI3K/AKT/mTOR axis appeared as the most prominent regulatory node, with multiple studies reporting reduced phosphorylation of pathway components accompanied by autophagy activation [29,36,38,40,41]. Additional mechanisms included ROS generation [42], Ca2+-dependent NMDA receptor signaling [41], and microRNA-mediated regulation [40], each feeding into autophagy-regulatory checkpoints (Fig. 2). Notably, although different MF modalities activated distinct upstream triggers [38,[40], [41], [42]], their downstream convergence on mTOR inhibition and autophagy-related protein expression [29,36,39] points to a shared capacity to restore impaired cellular quality control. The convergence on mTOR pathway modulation underscores the clinical promise of MFs as non-invasive alternatives to pharmacological inducers such as rapamycin. Unlike systemic drugs, which often produce off-target or metabolic effects [14,15], MFs interventions could provide organ- or cell-specific modulation while preserving systemic homeostasis.

The context-dependent nature of MF-induced autophagy was another salient finding. In neurodegenerative and stress models, MF stimulation promoted flux and contributed to neuronal protection and functional recovery [29,36,38]. Conversely, in cancer cells, MFs precipitated autophagy-associated cell death [40], reinforcing autophagy's dual role as both a survival and a death pathway. This dichotomy highlights the need to tailor MF regimens to disease context, aiming to promote cytoprotective autophagy in neurodegeneration and tumor-suppressive autophagy in malignancies.

The heterogeneity observed across the included studies can be explicitly categorized along three axes. First, MF intervention parameters varied widely in modality (rTMS, rotating fields, pulsed fields, power-frequency fields, radiofrequency exposures), frequency (1 Hz–935 MHz), intensity (20 mT–1.5 T), exposure duration (single sessions of minutes to chronic protocols of six months), and waveform. Second, model systems comprised six different cell types (mouse embryonic fibroblasts, human bone mesenchymal stromal cells, SH-SY5Y neuroblastoma cells, N9 microglia, primary astrocytes, and HT22 hippocampal neurons) and five distinct animal disease models (APP/PS1 AD mice, two-vessel occlusion vascular dementia rats, spinal cord injury mice, chronic unpredictable mild stress mice, and Lewis lung carcinoma-bearing mice). Third, outcome measures ranged from single-marker assessments to multi-marker panels with functional flux verification, making direct comparison of effect sizes across studies impracticable.

The concept of MF “dose” merits dedicated consideration. Unlike pharmacological agents, where dose is typically expressed as mass per unit body weight, the biologically effective MF dose is a multidimensional construct encompassing at minimum intensity, frequency, waveform, duty cycle, exposure duration, and inter-session interval. The existing literature has predominantly tested isolated parameter variations rather than employing factorial or systematic designs capable of revealing interactions among these variables. The observation that autophagy activation occurred at 0.5 T but not at 1.0–1.5 T in human mesenchymal stromal cells [41], and that both 1 Hz and 50 Hz protocols were effective in different models, argues against a simplistic linear dose-response and instead suggests the existence of discrete biophysical windows of efficacy. Future studies should adopt systematic dose-finding approaches, ideally incorporating response-surface methodology or factorial designs, to map the parameter space and identify optimal stimulation regimens for specific disease contexts.

A unifying mechanistic framework can be tentatively proposed. Regardless of their specific modality, MFs may share at least three proximal biophysical entry points: (i) perturbation of transmembrane ion channel gating, particularly voltage-sensitive Ca^2+^ channels, leading to altered intracellular Ca^2+^ dynamics; (ii) modulation of radical pair reactions, resulting in increased intracellular reactive oxygen species generation; and (iii) direct effects on protein conformation and membrane fluidity. These upstream perturbations subsequently converge on established autophagy-regulatory nodes. Specifically, Ca^2+^ influx can activate CaMKK-beta/AMPK, which inhibits mTOR; reactive oxygen species can activate AMPK directly and stabilize Beclin-1 complexes; and microRNA modulation (e.g., miR-486 suppression of BCAP/AKT) represents an additional regulatory layer. The net effect is convergent inhibition of the PI3K/AKT/mTOR axis, the master negative regulator of autophagy. While this integrative framework remains hypothetical and requires direct experimental validation, it provides a testable model for future mechanistic investigations and may explain how physically diverse MF stimuli elicit overlapping downstream molecular responses.

The translational relevance of these findings is considerable. In this regard, it is noteworthy that rTMS is already clinically approved for psychiatric and neurological disorders [44], establishing both a regulatory precedent and a technical infrastructure that could be leveraged for autophagy-targeted interventions. The ability to deliver temporally controlled and spatially focused stimulation may offer the possibility of tissue-specific autophagy modulation without systemic toxicity. This may be especially valuable in aging-related conditions, where gradual autophagy impairment contributes to multisystem dysfunction [10,11]. In addition, synergistic strategies appear promising; for example, rTMS combined with mesenchymal stromal cell transplantation enhanced autophagy induction more effectively than either approach alone [37], suggesting a future path for combinatorial therapies.

Nevertheless, several methodological considerations must temper interpretation. First, the small number of preclinical studies included in our systematic review and the heterogeneity of MF parameters precluded meta-analysis. The identification of only nine eligible studies reflects the nascent state of research at the intersection of MF biology and autophagy; our search spanned four structured electronic databases over a 15-year window using deliberately broad terms, indicating that the small yield is attributable to the novelty of this research area rather than overly restrictive inclusion criteria. Second, risk of bias assessments revealed variability in protocol design that could influence reproducibility. In some cases, co-interventions complicated attribution of effects to MF stimulation alone [37]. Third, while sham controls were generally included, most *in vitro* investigations relied on limited time points and lacked full flux verification; in addition, the frequent pairing of autophagy changes with favorable clinical outcomes in animal studies [29,36,38] raises concern about potential selective publication. Finally, a critical methodological caveat concerns the assessment of autophagic flux. Accordingly, only a minority of the reviewed studies employed gold-standard flux assays [41,42]. Increases in LC3-II or other individual autophagy markers cannot be equated with productive autophagic flux, as elevated LC3-II may equally reflect increased autophagosome formation or impaired lysosomal degradation. Indeed, the profile reported by Zhang et al. [39] – i.e., simultaneous LC3-II and p62 accumulation with mTOR activation – is more consistent with impaired degradation than with effective flux. In this scenario, the reliance on incomplete marker panels in several studies [39,43] limits the certainty with which autophagy activation can be claimed and represents a key area for methodological improvement in future work.

Based on the current evidence, three research directions appear most pressing. First, systematic parameter optimization studies are necessary to delineate dose-response relationships across frequencies, intensities, and durations in disease-specific contexts. Second, mechanistic investigations should clarify whether MFs act primarily through canonical signaling (e.g., mTOR suppression, calcium signaling) or also involve novel biophysical processes – including the use of magnetically-treated water [45,46]. Third, translational and clinical pilot studies should evaluate whether the preclinical findings on autophagy activation can be reproduced in humans, particularly by capitalizing on established technologies such as rTMS.

In conclusion, MF modulation of autophagy represents a potentially promising direction toward biophysical alternatives to pharmacological interventions. The precision, reversibility, and relative safety of MF stimulation could enable dynamic control of autophagic pathways in ways that systemic drugs cannot achieve. If limitations such as incomplete flux verification and methodological variability are addressed through systematic optimization and translational validation, MF therapy could become a valuable complementary approach for aging-related conditions – including neurodegenerative diseases, malignancies, and other disorders marked by impaired autophagy.

## Declaration of generative AI and AI-assisted technologies in the writing process

Perplexity was used during the preparation of this manuscript to refine grammar and language.

## Funding

This research received no specific grant from any funding agency in the public, commercial, or not-for-profit sectors.

## CRediT authorship contribution statement

**Enzo Emanuele:** Conceptualization, Data curation, Formal analysis, Writing – original draft. **Alejandro Santos-Lozano:** Data curation, Writing – review & editing. **Susana López-Ortiz:** Writing – review & editing. **Kayvan Khoramipour:** Writing – review & editing. **Celia García-Chico:** Writing – review & editing. **Simone Lista:** Data curation, Writing – review & editing. **Piercarlo Minoretti:** Data curation, Formal analysis, Supervision, Validation.

## Declaration of competing interest

The authors declare that they have no known competing financial interests or personal relationships that could have appeared to influence the work reported in this paper.

## Data Availability

Data sharing is not applicable to this article as no new data were created or analyzed in this study.
